# The Role of Civil Society Organisations in the Integration of Migrants, Refugees and Asylum Seekers in the Italian Labour Market

**DOI:** 10.1007/s11266-022-00489-0

**Published:** 2022-04-26

**Authors:** Mattia Collini

**Affiliations:** University of Florence (Università Degli Studi Di Firenze), Firenze, Italy

**Keywords:** Civil society, Migration, Integration, Italy, Refugees

## Abstract

In this paper, we address the role of civil society organisations (CSOs) in Italy with regard to the integration of migrants, asylum seekers and refugees (MRAs) in the labour market. The paper analyses the role played by CSOs in practice, looking at the dynamics of demand and offer of services through the perspective of both the CSOs and MRAs. To achieve this, we combine qualitative data from semi-structured interviews to CSO representatives as well as MRAs. Our findings point out that the fragmentation of the policy framework in terms of employment and integration, and an unfavourable legislation (above all, migration law) shape the kind of prevalent activities of CSOs and negatively impact the potential for integration of MRAs in the labour market. In general, much is left to the single CSO to fill in the needs of MRAs beyond minimal provisions established by law, with just asylum seekers and refugees having better opportunities and support. Furthermore, we can also observe how economic migrants generally tend to be less supported.

## Introduction

The role of civil society organisations (CSOs) in Italy and, more broadly, the ‘third sector’ has been the subject of different strands of the literature, as they constitute a pillar of the Italian welfare model in line with the southern European welfare model (see for instance Ferrera, 1996, 2001, 2005; Fazzi, 2013). Historically, civil society organisations have been involved in welfare politics in mutual accommodation with the state, with a substitutive role in providing basic public services. The expansion of the activities of the third sector in the past decades reinforced the division of labour between the State, which defines welfare policies, and CSOs providing the services (Ranci, 1994, 1999). Indeed, to this day, CSOs are still active as service providers, in particular at a local level, to provide assistance with accessing the welfare state system (Baglioni & Montgomery, 2020b).

In line with its key role in the welfare, the third sector has been widely acknowledged as crucial for providing assistance and integration of migrants (see, among others, Ambrosini, 2000; Santagati, 2004; Vellecco & Mancino, 2015). Over the years, a relevant literature made of journal articles, monographies, reports and various articles has emerged on the integration of migrants in Italy. However, as the broad concept of migration and integration of migrants is a rather complex issue, there is no single strand of the literature dealing with the topic of CSOs. Analyses and assessments of policies concerning the tools, paths and strategies of integration—including in and through the labour market—have been summarised by both collective and interdisciplinary research (Cerrina Feroni & Federico, 2018) and handbooks concerning the immigration law (Di et al., 2019). According to this literature, in general there is a gap between the policy rhetoric and the reality of immigration, and the most recent Italian policies respond more to the former instead of addressing the latter (ex multis Ambrosini, 2017). More recently, Campomori and Ambrosini (2020) have published an article analysing the critical aspects of the governance of asylum seekers’ reception in Italy and its dispersive multilevel approach. This literature is not focused on the role of CSOs, nor labour market integration, but it rather provides a more general overview. The topic of the labour market integration is instead present in scholarly works from different disciplines focusing on other aspects, which concern, in particular, education (Azzolini, 2015), employment related policies (Accorinti, 2017), the opposition to irregular/informal employment and labour exploitation (Chiaromonte, 2018; Sagnet & Palmisano, 2015) or reception services (De Petris, 2018) and the welfare rights of migrants (De Marcello & Lagravinese, 2015). Therefore, the picture that emerges is quite fragmented, and to our knowledge, there was no significant literature on the role exercised by civil society organisations in this field. Still, the role of CSOs is more prominent in different strands of the literature, focusing both on case studies on local integration processes (Bazurli et al., 2020; Bonizzoni & Marzorati, 2015) and on the broader role played at a national level by CSOs linked to the Catholic Church (Ambrosini, 2017, 2019). Valuable data and pieces of information regarding civil society organisations (CSOs) are also contained in research reports produced by associations and research centres (see, ex multis, Oxfam, 2016; Capitani, 2019; IDOS, 2019; ISMU, 2019) or government bodies (for instance Ministero Dell’Interno, 2017) and websites of the associations. On a comparative perspective, OECD reports analysed the role of CSOs in the integration of refugees and asylum seekers, although not necessarily focusing just on labour market integration, such as Galera et al. (2018a, b).

However, the most significant gap in the literature regards the role of CSOs in relation to the integration of migrants in the Italian labour market, with a relatively limited amount of scholarly research on this issue beyond specific case studies or non-scholarly works such as blog post and reports, or as a topic for several symposiums. Among the most recent contributions focusing specifically on Italy, ISMU published a comprehensive research paper on the socio-economic integration of refugees, focusing on practical experiences of NGOs (Sarli, 2019), which gives a clear representation of the vitality and practices of CSOs. However, due to chronological limitations, the study does not address the normative changes implemented in late 2018. Finally, the SIRIUS Project Italian national report (Maggini & Collini, 2019) provided a general overview of the role of CSOs in the labour market integration of MRAs, as part of a comparative study covering six other European countries (Numerato et al., 2019).

Building upon the findings of the SIRIUS research, which provided a first overview, this paper aims to contribute to fill the gap in the literature by providing an illustration of the role of CSOs in the integration of migrants, refugees and asylum applicants into the Italian labour market. What are the main barrier and enablers that hinder or favour a successful integration? What are the main challenges faced by CSOs operating in this field? What is the perception of MRAs with regard to the role of CSOs? These are the main questions we addressed during our research and fieldwork. In order to do so, we first present an overview of the role CSOs play in Italy, and particularly in support for migrants, refugees and asylum seekers, followed by the methodology employed in our analysis. The main section presents more in detail the activities of CSOs related to the integration of MRAs in the Italian labour market, based upon our findings, outlining the main barriers and enablers from the perspectives of both CSOs representatives and MRAs. Finally, we discuss our assessments, followed by a conclusive section which summarises the main findings and outlines the potential directions for further research.

### The Background: The Role of CSOs in the Integration of Migrants in Italy

In the space of a few decades, Italy, a country of emigration, turned into a destination for immigrants looking for a better life (1990s to the early 2000s). The arrival of economic migrants started to increase significantly in the early 2000s, which also saw a politicisation of the issue and the implementation of the first restrictive policies. Since the mid-2000s, Italy also saw an increase of refugees and asylum seekers, which culminated with the migration crisis of 2014. For a more detailed overview of the evolution of immigration in Italy, we can refer for instance to the works of Colucci (2018a, b) and Ambrosini (2013). The incorporation of migrants within the labour market is characterised by its complementarity with the labour market of Italians that often generates ‘ethnic specialisations’ (or occupational segregation) in low-skilled jobs often avoided by the natives. In this context, foreigners easily face discriminatory behaviours, widespread risk of informal employment and highly unstable jobs. This situation is further affected by an unfavourable policy framework regarding immigration and regularisation procedures.

Looking at third-sector organisations in Italy, we have a large non-profit sector, with many operating in the social sphere, where we have a prevalence of voluntary organisations, social non-profit NGOs (ONLUS) and social cooperatives (for more detailed information see ISTAT, 2019). Before the so-called migration crisis of 2014, Italian CSOs dealing with migration were mostly active in providing support to undocumented migrants, particularly in terms of access to healthcare and legal services against discrimination and exclusion (Ambrosini, 2013); or to promote integration and intercultural exchanges. Few were providing services targeted specifically at integrating migrants into the labour market, with language courses being some of the most diffuse activities, while job placement was left mostly to word of mouth, ethnic networks and for-profit agencies (for an overview of the policies of integration before the 2014 migration crisis see, among others, Campomori, 2008; Caponio & Zincone, 2011; Caneva, 2014). In parallel, while institutional actors were retreating from their responsibilities of international protection, CSOs often emerged as a substitute for asylum governance, as they kept on providing welfare services from below (Ambrosini & Boccagni, 2015; Bazurli et al., 2020).

The picture changed after 2014, with the proliferation of CSOs dealing with MRAs, particularly in the field of assistance and reception when, in order to cope with the new challenges of the migratory crisis, the Italian system of reception of asylum seekers was reorganised into two different tiers. The first-line reception was dedicated to the immediate aid and identification of asylum seekers, as well as initial assistance for the asylum application, but it lacked specific paths of integration.^1^ Afterwards, asylum seekers who did not have sufficient financial resources were transferred to second-line reception centres, which were managed by local municipalities within the national system of protection for refugees and asylum seekers (the SPRAR network). The 2015 reform also established the Centres of extraordinary reception (CAS), which were supposed to temporarily accommodate asylum seekers if there were no places available in other first-line governmental facilities and second-line SPRAR facilities. Despite being formally part of the first-tier reception, they were in practice a hybrid between first- and second-line reception centres. Their relevance within the reception system is underlined by the fact that CAS soon lost their extraordinary character, becoming the prevalent facilities for the long-term hosting of asylum seekers, in particular after the ‘Salvini Decree’.

This resulted into an increasing number of social cooperatives and other CSOs that activated ad hoc programmes for asylum seekers and refugees, often responding to public tenders. Such increase is particularly evident when we look at the numbers of the SPRAR programmes,^2^ which has existed since 2002: before the migrations crisis, they only involved a limited number of people—they hosted 1365 refugees and asylum seekers in 2003 and 3000 in 2010, compared to the over 20.000 since 2014, while the number of active projects was 134 in 2009 compared to 465 in 2014 (Ministry of Interior). In parallel, since 2015 CSOs have been managing the majority of CAS centres (Openpolis & Action Aid, 2020). Thus, CSOs were entrusted by public authorities with the task of carrying out not only assistance activities (for example, providing board and lodging), but also services aimed at favouring the integration of new arrivals, including inclusion in the labour market (for example language courses, job orientation activities, CV writing, training internships, vocational training). This largely remained the same also after the introduction of the so-called Salvini decree (Decree law no. 113/2018 on immigration and public security), although we assisted to a reduction in the number of subjects and personnel involved due to a significant decrease in funding and the reorganisation of the SPRAR system, which was renamed SIPROIMI. In general, we can divide the associations between those that deal with the reception system (CAS and SPRAR/SIPROIMI) and those that provide support for migrants outside the receptions system. These associations range from voluntary associations to intercultural centres, social promotion associations, social cooperatives and service cooperatives, providing a multitude of reception and integration services. As anticipated, they are not all are involved in supporting the integration of MRAs in the labour market, although we shall not discard the relevant role of those that through formal and informal activities indirectly contribute to the integration of migrants in the labour market (i.e. language courses, legal counselling).

Indeed, the level of offered support highly varies between first-tier (CAS) and second-tier (SPRAR/SIPROIMI) reception, where the former provides only a limited amount of support, generally on a voluntary basis, and the latter provides a full range of services for social and economic integration. Considering that around 80% of those in the national reception system are hosted in CAS centres, the general outlook is not favourable. Furthermore, there is ample variation in services provided in different regions and even between different CSOs (Cuevas, 2020). For CAS centres, there is no uniformity of services and even SPRAR/SIPROIMI projects, despite having a wide range of statutory services to provide, have a high degree of autonomy on how to implement them. Thus, the Italian system of reception and integration has been defined as a sort of ‘lottery’ for arriving migrants (Oxfam, 2017). This diversification of services and experiences can be linked to two main causes: the legislation that regulates reception and integration, and the lack of national policy frameworks, which results in policy fragmentation and is aggravated by limited coordination among the several actors involved in the governance of the phenomenon. This adds up to the decentralised nature of the Italian system of government, which delegates to the regional government competences in the fields of labour and professional education. On the other hand, this leaves some leeway to regional government to fill in the void left by the national legislation, if they have the political will to do so, which adds up to the fragmented nature of the system.

At national level, when we deal with labour market integration of MRAs in Italy, the most prominent actors are faith-based organisations along with non-profit organisations for international cooperation and NGOs. Several organisations are part of international umbrella organisations or have developed projects/partnerships abroad and operate at local level through their local branches, delivering a multifaceted basket of services and activities, ranging from mere reception activities to socio-economic inclusion services (language courses, job counselling, training internships, etc.). Beneficiaries are both economic migrants and asylum seekers/refugees, but in recent years these organisations have been particularly involved in the management of both first-level and second-level reception centres, in line with the general trend. In other cases, we can have organisations such as the ISMU Foundation that are working to create networks and promote the best practices to facilitate the labour market inclusion of MRAs, with a bottom-up approach that starts from the experiences of the civil society (Sarli, 2019).

Instead, most CSOs dealing with the labour market inclusion of MRAs operate at the local or regional level within the SPRAR/SIPROIMI system or in the first-tier reception system of the CAS (extraordinary emergency centres). Labour market integration is also promoted through partnerships, activated by the host structures or organisations that promote specific projects, with employers and non-profit organisations working together, such as the associations of the ‘Rete migrazioni e lavoro’, and those involved in national and international projects such as LABOR-INT and ‘Next’ (New Experiment for Training). Other association active in the management of CAS also created networks in order to secure resources and provide some form of support for the economic inclusion of their guest, such as the ‘Bonvena’ network operating in Northern Italy (Sarli, 2019). Finally, we can mention other experimental programmes, such as those promoting the integration of refugees and asylum seekers in remote areas. Those programmes try to counter the depopulations of such areas and boost local socio-economic development through the integration of migrants, with CSOs often acting as mediators between migrants and local communities, although they had only achieved mixed success in their past (Galera et al., 2018a, b).

## Methodology

Our research combines desk research (findings from the literature and online sources) with qualitative interviews data from CSO representatives as well as current and past beneficiaries of integration services. Data from semi-structured interviews allows exploring the views of CSOs’ representatives on migrants, refugees and asylum seekers, their employability and integration potential in recent years, particularly after the migration crisis. Looking at the issue also through the migrants’ lenses conveys a more complete picture, underlining the real needs and dynamics for their integration in the labour market. Two separate sets of question were prepared for CSOs representatives and MRAs. All interviews have been conducted between May and November 2019. The recruitment of informants largely relied on the collaboration of the various stakeholders encountered during the work for the SIRIUS project, as well as personal contacts, and ‘snowballing’.

Given the rather fragmented nature of the Italian system, in order to get a comprehensive view of the types of association and their geographical distribution, we tried to include organisations operating in different socio-economic contexts. However, we could not ensure an equal number from each area, with a prevalence of Central and Northern Italy (Lombardy, Emilia-Romagna and Tuscany)—which is an area with a traditionally higher social capital and employment rate—and with just one NGO operating at a local level in a predominantly agricultural region in Southern Italy (Apulia). Nevertheless, we still managed to present a broad range of experiences and practices that depicts an informative picture of the role of CSOs in Italy. In total, for this research we employed data from 23 interviews with CSO members and 24 interviews with MRAs. The first are mostly operators/workers of associations, while in some cases also founders or presidents/directors of the association itself. With regard to the type of organisations, we reached faith-based associations (3), social cooperatives (12), voluntary organisations (4) and other kinds of NGOs or foundations (4) involved at various level in the integration of MRAs. The main target group of the associations we interviewed are asylum seekers and refugees. Two CSOs have as target groups women victims of human trafficking and exploitation. Economic migrants are not the main target, albeit some CSOs deliver some labour market integration services to foreigners in general, without having a specific target group. For the recruitment of MRA interviewees, we focused on people who have arrived in Italy in the last decade, with a prevalence among our interviewees of asylum seekers and refugees hosted in reception centres (15), while just four are economic migrants. Concerning gender, of the 24 interviewees only five are women. Most of them (20) are coming from sub-Saharan Africa (5 from Ivory Coast; 4 from Nigeria; 3 from Guinea; 2 from Ghana, Togo; 1 from Mali, Senegal, Sierra Leone and Somalia), two from Northern Africa (Egypt, Morocco) and two from South Eastern Europe (Bulgaria, Albania). The large number of persons coming from sub-Saharan Africa is consistent with the nationalities declared by those people who arrived in Italy through the so-called Mediterranean Route, which also represents the majority of refugees and asylum seekers arriving in Italy since the beginning of the migration crisis. More details about our interviewees can be found in “Appendix” for both CSOs (Table 1) and MRAs (Table 2).

## The Role of Civil Society Organisations

As we have previously seen, CSOs are highly involved in the socio-economic integration of MRAs in Italy. Here, we will assess the direct experiences of CSOs with regard to the supply and demand of services, in conjunction with the main obstacles to overcome in order to promote a successful integration in the Italian labour market. In parallel, we also assess what is the perception of MRAs with regard to the role of CSOs.

### Identifying Barriers and Enablers

In the previous section, we have briefly mentioned the kind of activities CSOs are mostly involved into, such as the reception service and its related needs. The analysis of interviews with CSOs representatives confirms their relevance in the reception system, but also supplements the existing literature, allowing to assess in detail their specific role in regard to LMI. We can identify several fundamental services provided by CSOs to facilitate the integration of MRAs in the labour market, particularly those involved in SPRAR/SIPROIMI projects: (1) language courses; (2) cultural mediation; (3) identification of skills and inclinations; (4) voluntary work; (5) training and education (in particular middle school degrees); (6) education on the rights and duties of workers and the characteristics of the Italian labour market (also in cooperation with trade unions); and (7) training and internships. Cooperation among associations, participating in national and, in a few cases, international networks, is also mentioned by interviewees as an important facilitator, allowing to exchange experiences and best practices and deliver positive outcomes in terms of integration outcomes (CSO interviewees 6, 9, 21, 22). Some associations see their role as facilitators: *‘I am not a centre for employment. We do not assess skills, we rather offer guidance and assistance for job search […] The reception centre must be like a gym where one prepares to be autonomous’* (CSO, Interviewee 12). Indeed, an extremely relevant point for a complete and successful integration that emerges from our interviews (CSOs Interviewee 6, 10, 11, 19), is to prepare MRAs to be autonomous once out of the programme, not only thorough a care approach but by pursuing the empowerment and autonomy of people. In this regard, the creation of personalised paths with an ‘holistic approach’ towards migrants’ labour autonomy has been proved to be effective. This is achieved on the one hand by working on the acquisition of personal and professional skills on the one hand and on the protection of the worker on the other, focusing on work safety, workers’ rights, how to recognise and avoid the ‘black’ and ‘grey’ labour. In other words, providing the necessary tools to fight exploitation.

A specific set of barriers to the integration of MRAs to be emphasised are those linked to the legal and policy framework. According to some interviewees, the reception system as a whole is not well organised*: ‘it is left to the associations of the private social sector […] from the point of view of the labour market inclusion policies there is no real systematization; for example, there is no systematization of the Italian language courses for job market orientation, there is no system that favours the match between labour supply and demand […] the role of informal networks to find a job is still prominent and this has a greater negative effect on migrants who have less extensive networks when they arrive … in the past, economic migrants entered through a series of family networks that for better or for worse provided information … now for asylum seekers this network is missing’* (CSO, Interviewee 8). Notably, the interviewee outlines some limits of the Italian employment policies (i.e. lack of proper supply and demand matching), as well as the structure for the Italian labour market, which disproportionally impact the integration of migrants.

Furthermore, the consequences of the new legislation introduced after the ‘Salvini decree’ were detrimental also for the integration of asylum seekers. Among the most critical issues reported we have the legal status of beneficiaries, *‘it makes many migrants illegal immigrants. Because of the abolition of humanitarian protection, many cannot convert their residence permit into a work permit’* (CSO, Interviewee 6), and the shifting away from diffuse reception: *‘the new decree favours big first reception centres, which are cheaper, but have a greater impact on local communities […] such centres often provide only basic activities, such as room and board. Conversely, SPRAR second reception centres are downsized: they are only for unaccompanied minors and refugees’* (CSO, Interviewee 3). The cut in funding provided by the public tenders and the removal of the few provisions for integrations services and support for CAS centres had negative consequences both for people working in the sector and for asylum seekers, who were excluded from the SPRAR system. *‘Italian language courses [in CAS] are no longer obligatory, there is a clear plan to destroy the paths of integration dictated by political motivations. With the new tenders, for large-scale economies, people are concentrated in big reception centres by reducing the services just to save money. We are speaking of an operator for every 50 people: it is impossible to provide real integration paths’* (CSO, Interviewee 19). In short, the new situation for CAS operator can be well summarised by the word of Interviewee 22 ‘*Nowadays, if a migrant is a SIPROIMI beneficiary, he can access a series of services, otherwise they have nothing. There is almost nothing for asylum seekers [coming from the State]. Institutions just now are opening calls for some projects and funding, but very few [and with limited resources]. Before it was possible to offer services’.*

Among the other most common problems, we have the recognition of the hard and soft skills of migrants when they try to enter the labour market. Due to their very different backgrounds, and often lack of formal education, it is necessary to offer training even to people with a previous professional background, particularly as in Italy several professions, even manual labour (i.e. construction, mechanics), have to follow different rules, procedures and regulations. In other cases, there are issues with the recognition of formal qualifications, particularly high school and university degrees, that pushes CSOs to provide support for education to obtain Italian degrees.

The socio-economic context where the CSOs are operating is also a relevant factor for the successful integration of migrants in the labour market. The best results are achieved when CSOs are offering labour market integration activities targeting the needs of the local economic context (CSO Interviewees 11, 13, 18). Several CSOs operating SPRAR or even CAS are thus trying to provide migrants with training and internship in line with the labour demands of the territories such as the logistic or food industries in the northern province of Parma, where these sectors are particularly developed, or health and safety courses for agricultural workers in the southern province of Andria, which relies mostly on agriculture. It also may not come as a surprise that CSOs report to have much better chances to integrate migrants in context with lower unemployment levels and social capital, where they can create virtuous relationships between associations, local administration and private companies.

### The Perspective of MRAs: Not All Migrants are Equal

The experiences of MRAs with CSOs and their services can be quite different according to their background and status. Thus, we have first to distinguish between economic migrants on the one hand and asylum seekers/refugees on the other hand, and subsequently between asylum seekers and refugees that have been hosted in first (CAS)- or second-tier (SPRAR) reception centres.

Economic migrants are less likely to interact with CSOs, although there is a certain variation in terms of experiences with CSOs. Among our interviewees, on one extreme there are those who found jobs through friends and by relying on themselves, without receiving any support by CSOs. In particular, an interviewee points out that for economic migrants or for those who enter through family reunification, there is no support either by public institutions or by CSOs: *‘I would have liked to have more support, no one informed me on how to find a job, no one offered language courses, *etc*.’* (Migrant, Interviewee 24). On the other extreme, we find a Moroccan woman fully integrated into the Italian society in terms of both work and social capital. She is currently working at an immigration desk managed by a cooperative, while previously she actively participated in a huge variety of civil society organisations and volunteering activities that helped her get ‘where she is now’. In an intermediate position, we found a young man from Nigeria, who recognises the help received by a job counsellor at the immigration desk, managed by a CSO, that highlights the importance of the collaboration between public institutions and CSOs to deliver integration services. CSO can also play a crucial role for migrants in peculiar situations, such as for victims of human trafficking and exploitation, to favour their integration into the Italian society (Migrant Interviewee 6).

Asylum seekers and refugees are in a very different situation: all of them, indeed, were obligated to deal with CSOs, given that they are/have been hosted in reception centres, which are generally managed by CSOs. In this regard, there is a substantial difference between second reception centres of the SPRAR system and first reception CAS centres. Refugees who passed through the SPRAR system are reporting mostly a positive opinion about the help received by CSOs, as they can benefit from a range of dedicated services for social and economic inclusion, such as job searching orientation, vocational training and inclusion into companies through training internships or job grants. In general, they stress the usefulness of language courses and training internships: *‘they helped me a lot, giving me an accommodation, food, documents, everything […] [they] paid for a training internship within the cooperative where I’m currently employed. After the internship, I got a short-term contract and then a permanent contract’* (Refugee, Interviewee 5). Yet, some lament the lack of preparation towards an autonomous life once out of the system, after having been dependent on the operators, particularly about finding accommodation: *‘operators tend to assist you in everything without leaving you any autonomy. But when you leave the centre, you are not very able to get away with it alone. In fact, you move from 100 to zero in terms of support’* (Refugee, Interviewee 1).

Contrary to what is granted by SPRAR centres, CAS centres do not have to provide any services in terms of labour market integration. The type and quality of services offered depends on who manages them, often leaving the migrants hosted there to rely on their own, or on the help of informal networks of hosts or former hosts. *‘According to my experience, it was easier to find work through friends and migrant networks, rather than through associations […] out of three job opportunities I had, two were through friends. Even the third […] I finally found it through an acquaintance’* (Refugee, Interviewee 8). Nonetheless, migrants hosted at CAS are expressing the need for a job and have many reasons to do so going from the need to achieve independence, receive a working residence permit, but also to preserve their dignity. ‘*I want to work, I can do any kind of work*’ (Refugee, interviewee 2). Indeed, work and job placement are fundamental in the integration process of MRAs: if skill recognition is often a barrier, vocational training and skill enhancement are recognised as major enablers, despite often resulting in jobs not in line with one’s past qualification (for those who had one). This is accompanied by the fundamental contribution of language courses and cultural mediation.

In general, we can summarise the most important services requested by migrants as: (1) language courses; (2) integration paths to achieve an autonomous life in the Italian context/society; (3) trainings and internships; (4) general education; an d(5) legal assistance. These are largely in line with the perception of needs and services offered by CSOs, although often in too limited numbers. Still, in the eyes of MRAs it is not always clear what CSOs can do for them and what they cannot do (e.g. CSOs are occasionally confused with employment centres); thus, sometimes they make requests that cannot be satisfied. Interview data with MRAs concur that, regardless of their background, getting a job is one of the first goals for migrants arriving in Italy, usually any kind of job regardless of their previous qualifications, which also shows a large degree of flexibility. This also includes asylum seekers who did not originally choose Italy as their destination. Consequently, the main expectations they have from CSOs, if they interact with one, are to be supported in finding a job. However, not all CSOs have the capacity and possibilities to meet such expectations.

### Filling the Gap? Dynamism and Limits of CSOs

We have seen that CSOs may provide a wide range of services to support MRA’ integration in the Italian labour market, depending on the kind of associations and what kind of programme they are involved into, if any. Indeed, there is a wide difference between the range of services that first- and second-tier reception centres provide for asylum seekers and refugees hosted, which is strictly correlated to the policies that regulate reception and integration. However, there is also another aspect that we shall assess, and it is the role of CSOs in how they provide services considering the legal framework, the direct experiences of CSOs and the demands of MRAs. Indeed, we observed that several CSOs are trying to offer more services than those mandated by law, or more targeted and comprehensive services than what established in the tenders. ‘*The message that has always come to us is: ‘we only do what is required by the prefectures: if we manage to integrate people, that is better, but it is not mandatory*’ […] *fortunately we are all young people with clear beliefs and therefore we try to integrate them*’ (CSO, Interviewee 19). This is also consistent with other case studies such as Bazurli et al. (2020).

The additional effort to provide targeted and effective labour market integration support was expressed in a particularly vocal way by an interviewee who is also a social operator in a CSO involved in SPRAR/SIPROIMI projects in Northern Italy. ‘*We become agents to promote dialogue and contacts between the workers [the asylum seekers and refugees hosted by the association] and the employers. We also involved other social agencies like trade unions in the process. All this was not requested by the SPRAR/SIPROIMI projects. Our work orientation and formation area in particular… We are working above the lines… If we had to offer only what is required by the project, we would just offer language courses and job orientation classes. I would not offer legal advice, I wouldn’t teach how to deal with contracts, I wouldn’t involve trade unionists…’* (CSO Interviewee 9). Despite all, there is a sense that individual efforts promoted by CSOs are not enough to overcome the legislative and policy barrier, just to mitigate it. ‘*We did go beyond the requirements of the ministry. I could have done only training courses and language classes, no-one obligated us to act ourselves. Still […] we are not able to totally fill in the legislative gap’ (CSO Interviewee 9).*

Such an approach is even more relevant for those involved in first-tier reception centres where there is no specific requirement for labour market integration, but many CSO still tried to provide some support to meet the needs of their hosts. This was clearly stated by a representative of an association running both CAS and SPRAR/SIPROIMI projects. *‘Before the Salvini Decree we were having a project for labour market integration and orientation [in our CAS]. We were keeping one euro a day from the daily sum for each guest […] It run approximately 2015–2018. This all changed after the Salvini decree… We were working with the territories, providing internships, training, and provided formation to work […] in order to find a match between employers and job offering. Scouting first, then selection and matchmaking. Our Italian language courses were structured differently than required and they were tailored to allow an empowerment process. […] The trainings were chosen based on an exchange of experience between employers, associations, and trade unions, taking into account the needs of the territory. Now it is just not possible, we can offer it only for our SIPROIMI projects’* (CSO Interviewee 22). The interviewee also highlights the consequences of the ‘Salvini Decrees’, when such practices became increasingly difficult to be provided due to the reduction in funding and statutory services to be provided in CAS. However, we also have opposite examples, where other associations challenged the new law: *‘[our association] did not throw out a single person hosted with a humanitarian protection permit*^3^*in spite of the new legislative changes. … We continued offering them all the same as before’* (CSO Interviewee 9). However, this is a course of action that can be taken only by those associations that possess or can access the required resources.

A final element that was present in the words of our interviewees is the role of providing tailored services that can benefit from a cooperation among various stakeholders in accordance with the specific socio-economic context. Some of them also tried to create a so-called third-tier reception for refugees and beneficiaries of humanitarian protection, dedicated to transition them towards a complete autonomy, focused on labour market integration, after an often too short period in the SPRAR/SIPROIMI system, or a lack of integration support in CAS. Unlike first- and second-tier receptions, these initiatives are not formalised and created on discretional and voluntary basis by those involved and they represent a clear example of the activism and dynamism of the Italian civic society (Sarli, 2019). Among the most relevant examples, we can mention CIAC, which now manages a network of services throughout the province of Parma: from migrant orientation desks for the municipalities, acting as a link between the various public services, up to social housing for people leaving the SPRAR system but who have not yet consolidated a stable job and economic position. Such approaches, providing additional services through project-based resources, are now paramount for those associations that want to keep providing additional integration support. Nevertheless, beside the relevant number of virtuous examples, there are still numerous CSOs that only provide what prescribed by law and in the tenders for CAS and SPRAR/SIPROIMI projects.

## Discussion

In Italy, CSOs have a long history of being involved into the integrations of migrants, and our research shows how they are also playing an essential role in relation to the integration of MRAs in the labour market. However, the role of CSOs in the integration of migrants into the labour market is much more significant for refugees and asylum seekers, which correspond to a change in the influx of extra EU migrants. The number of non-seasonal economic migrants arriving in Italy sharply declined in the past years due to a restrictive legislation. This became particularly evident with the 2014 migration crisis, when we witnessed a proliferation of associations involved with migrants, responding to the emergency and the consequent legislative changes and inflow of public resources. CSOs, just as in many sectors of the welfare system, are providing services and are being contracted by the state in a way that is somewhat comparable to what was already identified by Ranci (1994) a quarter of century ago, where the State delegates to NGOs, except that now we assist to a proliferation of actors in a more liberalised world. Thus, it appears that the role played by CSOs with regard to labour market integration of MRAs is connected to the legal framework and policy paradigms. Indeed, the influence of the legal and policy framework has been identified by several stakeholders as one of the main barriers for the integration of MRAs. Legislation and policies had a relevant impact in shaping the current role of CSOs as they were entrusted by the State with managing a prominent part of the system of reception and integration programmes, responding to public calls and tenders to provide CAS and SPRAR/SIPROIMI services. In particular, SPRAR/SIPROIMI projects are required by law to be run by non-profit organisations in cooperation with municipalities, which contributes to CSOs being mostly active at the local level rather than the national level.

With regard of the demand and offering of services, most activities are dedicated to support the integration in the Italian labour market of asylum seekers and, above all, refugees, along with unaccompanied minors hosted in SPRAR/SIPROIMI centres, which, in terms of numbers, constitute a minority of those hosted in the national reception system, and more generally of extra-EU MRAs present in Italy. This is also reflected by the existing literature on the subject, which concentrates on policies and realities concerning the integration of refugees and asylum seekers. The absence of a comprehensive national framework clearly plays a key role in determining the successful integration of MRAs into the Italian labour market. In general, CSOs have a negative outlook on the outcomes of current legislation approved in late 2018, which limited the beneficiaries of the SPRAR services only to unaccompanied minors and people who have been granted international protection. This was accompanied by a reduction in funds and requirements for the first-tier reception centres (CAS), which implied also the termination of limited integration services such as language courses, cultural mediation and, where present, basic labour market orientation. Therefore, several associations, in particular social cooperatives, denounced that they would be forced to terminate their involvement in the reception. The main reasons have been attributed to both a reduction in funding and the refusal of several CSOs to operate reception centres that provide only minimal lodging facilities making them de facto managers of a sort of detention centres rather than social operators aiming to provide integration perspective to migrants (Openpolis & Action Aid, 2020). This left mostly larger entities able to sustain the conditions of the tenders for the management of the reception for asylum seekers, which have been identified as the least efficient in terms of a successful integration (Oxfam—In Migrazione 2019). This holds particular significance as CAS are now hosting an even higher proportion of asylum seekers. Such developments could be mitigated only in part by CSOs that could find the necessary resources to provide even minimal labour market integration support through external projects.

Still, not all CSOs are active in the field of reception and assistance to refugees and asylum seekers, or not exclusively. Among our sample, we find some organisations that also provide a range of services, often in the forms of help desks, to foreigners in general, without specific target groups. Nonetheless, economic migrants are generally not involved with CSOs for labour market integration. Among economic migrants, those who turn to CSOs are either longer-term migrants, or looking mostly for language training or administrative assistance, while when looking for jobs they might usually resort primarily to informal networks, followed by ‘ordinary’ channels for job searching and placement such as public employment centres, specialised helpdesks (where present) or private agencies (see also Collini & Pannia, 2020). On the other hand, CSO are also crucial in terms of supporting the most vulnerable of migrants, such as women victims of human trafficking, or exploitation. Another important thing to mention is how some of the services, workshops or activities organised in particular by intercultural associations represent a way to overcome cultural boundaries and be integrated into society, particularly in case of women (CSO Interviewee 17), which can indirectly favour a process of empowerment and their integration into the labour market.

In general, the help received by CSOs is appreciated by the MRAs interviewed, especially by those who arrive without a family network. In parallel, over time since the beginning of the so-called migration crisis, CSOs’ operators acquired a professionalism that allowed them to respond more efficiently to the needs of the migrants, also by employing former beneficiaries. Indeed, the ‘booming’ of the reception system after 2014 initially forced several NGOs and cooperatives to recruit personnel with little practical experiences. This confirms CSOs as those more aware and aligned to the actual needs and issues of migrants among the various stakeholders (Collini & Federico, 2020), which is also reinforced by the findings of the SIRIUS research (Baglioni & Montgomery, 2020a; Numerato et al., 2019). That said, however, not all CSOs’ services are considered as being particularly helpful in order to find a job: networks of friends, relatives and fellow citizens are often considered more important.

A final element that emerges from our research is that creating successful paths of integration for MRAs in the labour market is linked to the dynamism and involvement of civil society at a territorial level, and the capacity to create a synergy among all the stakeholders, particularly local economical actors. Indeed, several of the most active and successful stories/practices are from areas with a well-developed economy and a tradition of highly developed public services and social capital (i.e. Emilia-Romagna, Lombardy) rather than agricultural-focused areas or areas with high rates of unemployment (i.e. most areas in Southern Italy), although notable exceptions exist. Another major enabler can also be considered the creation of personalised paths that allows a proper transition from the centres to the ‘real world’. This should be part of a process of empowerment to avoid the sensation of abandonment once out of the programme, as reported by an interviewee, and is in fact the guiding principle of several CSOs.

## Conclusions

In conclusion, we can summarise the role of CSOs with regard to the integration on MRAs in the labour market and integration in general in Italy, as largely service-oriented, centred on the reception system of refugees and asylum seekers. CSOs constitute the core of this system, where much is left upon them to fill in the needs of MRAs beyond minimal provisions established by law with just refugees and unaccompanied minors having better opportunities and support compared to asylum seekers hosted in first-tier reception facilities. CSOs generally well understand the needs of migrants, and this is largely confirmed by the MRAs interviewed who had experiences with CSOs. Based on the feedback of both CSOs and immigrants who have successfully integrated, the most important step to be taken would be to create virtuous cooperation between the public and CSOs, along with local stakeholder, to create tailored paths of integration using a ‘holistic’ approach. Indeed, we have virtuous examples of cooperation and successful experiences, but these are generally scattered and isolated, without a homogeneous diffusion across the national territory, and the most successful programmes are involving only a minority of the migrants. This can be generally linked to a lack of national coordination in terms of integration programmes, paired with strong decentralisation in the field of employment policies, that can be considered among the main reasons behind such fragmented and mixed results, alongside unfavourable normative and migration policy frameworks, as well as the adverse political context present at the time.

This is one of the first scholarly works to address the role of CSOs while also covering the initial consequences of the ‘Salvini decrees’ with a focus on labour market integration, and as, such we are aware its limitations, which are often linked to the still largely exploratory nature of the base research. The first limit is the timeframe of our research: our data do not allow to cover in full the consequences of the new policies, particularly with regard to the direct experiences of asylum seekers and former beneficiaries of humanitarian protection, as all the interviews to MRAs were conducted before the legislative and policy changes of late 2018 fully took effect. This leaves space for further research that address more in detail the impact of the post-2018 policy and normative framework on both the activities of CSOs and the opportunities for a successful integration of MRAs, with a focus on the consequences of the ‘Salvini decree’, and the new reform approved in late 2020 that is supposed to replace it. Another possible direction that should be explored is the role of the local socio-economic context on the supply of services and opportunities for migrants, which was just briefly mentioned in our research, and was limited by the territorial coverage of our fieldwork. In this regard, it would be particularly interesting to conduct a comparative research to assess the role and activities and CSOs, as well as the MRAs perspectives in different contexts (i.e. industrialised/rural; highly/limited development of civic culture; low/high unemployment). Finally, it would be extremely useful to assess the impact of the COVID 19 crisis and how it affects CSOs activities and prospects for the labour market integration of MRAs.

## Appendix

See Tables 1 and 2.

**Table 1:** Summary of CSO representatives interviewed

Identifier	Function/role	Type of institution
CSO 01	Member of the department for orientation, training and employment	Religious organisation
CSO 02	Work orientation desk for both asylum seekers/refugees (at CAS centres) and economic migrants	Social cooperative
CSO 03	Manager of a SPRAR project	NGO
CSO 04	Member of the local secretariat with responsibility for immigration issues	Social promotion association
CSO 05	Project manager, scientific coordinator and management of international projects	Non-profit private foundation specialised in job placement
CSO 06	Coordinator of the reception service for migrants	Religious organisation
CSO 07	Vice-president	Cleaning services cooperative
CSO 08	Project manager in charge of projects for the employment of disadvantaged groups in Italy	Non-profit organisation for international cooperation
CSO 09	Coordinator for work orientation and formation/education	Social cooperative
CSO 10	Responsible for the integration area within a SPRAR project	Social cooperative
CSO 11	President/founder	Social cooperative
CSO 12	Responsible for the integration area (training and employment) within a SPRAR project	Voluntary association
CSO 13	Operator in charge of migrants' integration	Social cooperative
CSO 14	member in charge of the work orientation service and language school	Religious organisation
CSO 15	Operator for the reception facility for women victims of trafficking and exploitation	Voluntary association
CSO 16	Social worker in charge of welcoming and integration of women victims of trafficking, member of the Board of Directors	Social cooperative
CSO 17	President/founder	Voluntary association/intercultural centre
CSO 18	President	Social cooperative
CSO 19	psychologist, operator in charge of education programmes and job placement	Social cooperative
CSO 20	Operator	Social cooperative

**Table 2:** Summary of MRAs interviewed

Pseudonym of Interviewee	Classification	Age	Gender	Family status	Country of origin	Migration year	Education
MRA 1	Refugee	34	Female	Divorced with children	Somalia	2008	Tertiary
MRA 2	Refugee	23	Male	Single	Ivory Coast	2016	Primary
MRA 3	Economic migrant	41	Female	Married with children	Morocco	2007	Secondary
MRA 4	Humanitarian protection	42	Female	Traditionally married (not officially) with children	Ivory Coast	2016	Illiterate
MRA 5	Refugee	19	Male	Married with children	Ghana	2015	Primary
MRA 6	Victim of human trafficking	33	Female	Married with a kid	Bulgaria	2008	Secondary
MRA 7	Economic migrant	33	Male	Married with a kid	Egypt	2010	Tertiary
MRA 8	economic migrant	33	Male	Married	Nigeria	2012	Tertiary
MRA 9	Asylum seeker	20	Male	Single	Ivory Coast	2016	Koranic school
MRA 10	Asylum seeker	22	Male	Single	Togo	2017	Secondary
MRA 11	Asylum seeker	24	Male	Single	Togo	2016	Secondary
MRA 12	Asylum seeker	23	Male	Single	Nigeria	2016	Secondary
MRA 13	Asylum seeker	18	Male	Single	Guinea	2017	Primary
MRA 14	Asylum seeker	21	Male	Single	Sierra Leone	2017	Secondary
MRA 15	Asylum seeker	22	Male	Single	Senegal	2016	Secondary
MRA 16	Asylum seeker	22	Male	Married	Ivory Coast	2017	Koranic school
MRA 17	Asylum seeker	31	Male	Single with a kid	Ivory Coast	2016	Secondary
MRA 18	Asylum seeker	38	Male	Married with children	Nigeria	2017	Primary
MRA 19	Asylum seeker	20	Male	Single	Guinea	2017	Primary
MRA 20	Asylum seeker	25	Male	Married with a kid	Guinea	2017	Primary
MRA 21	Asylum seeker	31	Male	Divorced	Nigeria	2016	Illiterate
MRA 22	Asylum seeker	36	Male	Widow with children	Ghana	2017	Illiterate
MRA 23	Asylum seeker	30	Male	Single	Mali	2017	Secondary
MRA 24	Economic migrant	33	Female	Married with children	Albania	2009	Tertiary

## Footnotes

1 The first-line reception system comprised the so-called hot spots: CPSA (Centres of First Aid and Reception), CDA (Reception Centres), CARA (Reception Centres for Asylum Seekers) and CAS (Centres for extraordinary Reception).

2 The Protection System for Asylum Seekers and Refugees (SPRAR) was created by Law No 189/2002 and is made up of the network of local institutions that implement reception projects for asylum seekers and refugees. The system is financed by the National Fund for Asylum Policies and Services, managed by the Ministry of the Interior. It is generally composed by small structures hosting a limited number of people according to a principle of ‘diffuse reception’ (accoglienza diffusa). In 2018, SPRAR was renamed SIPROIMI (Protection System for Beneficiaries of International Protection and for Unaccompanied Foreign Minors) and SAI (Reception and Integration System) in 2020.

3 This is a special kind of temporary residence permit which could be granted for humanitarian reasons, abolished in 2018 by the Salvini Decree and reintroduced in 2020.
